# How to Secure X-Ray Protection

**Published:** 1920-05

**Authors:** H. T. Fischer


### HOW TO SECURE X-RAY PROTECTION

The subject of X-ray protection can not be covered too thoroughly. Those who understand the peculiar burning effects of the X-ray have for years been expounding "safety first," and to remain free of ill effects while actively engaged in X-ray work means simply proper and adequate protection.

Years ago the operator, knowing practically nothing of the dangers of the ray, held his tube in a small wooden clamp with no protection whatever. Truly we have made progress, and at least know the fundamental principles of the ray and its action, and use all manner of protective devices. We have the leaded rubber tube shield, leaded glass X-ray tubes, and leaded glass protection bowls for surrounding the tube, all of which are effective and provide protection to a certain degree.

There are favorable arguments for all of these appliances, but if you desire real protection you should not rely on any one alone. Don't overlook the fact that the X-rays diminish inversely as the square of the distance increases.

The fact therefore clearly presents itself that working back of a screen at a distance from the tube lessens the danger to practically the zero point. Operating a unit type of equipment without some protective arrangement is much more dangerous than when the X-ray tube is held in an individual tube stand some few feet away.

A lead protection screen of sufficient size that the entire body is properly shielded, and covered with lead not less than one-sixteenth inch thick, should be employed when films or plates are made every day; or it might suffice to say, when the X-ray tube is used at least six times in any one day.

The trend to-day is to make X-ray units with adequate leaded glass protection, in addition to whatever is used to cover the X-ray tube itself.

It is safe to add that with the knowledge gained from past experience no X-ray burns or dermatitis need be feared, if the operator will only consult the equipment manufacturer to ascertain just how much protection is provided on his particular outfit, and when convinced that that protection is adequate, and not before, to purchase. A few dollars spent to-day for a well-made protection screen will certainly afford the operator peace of mind, and the knowledge that he is taking absolutely no chances with what we all know is a powerful, dangerous *thing,* and knowing that he is protected, will enable him to render better services to his patients by making radiographs wherever indicated.—H. T. Fischer, in *Dental Health.*

